# TudS desulfidases recycle 4-thiouridine-5’-monophosphate at a catalytic [4Fe-4S] cluster

**DOI:** 10.1038/s42003-023-05450-5

**Published:** 2023-10-27

**Authors:** Jonathan Fuchs, Rapolas Jamontas, Maren Hellen Hoock, Jonathan Oltmanns, Béatrice Golinelli-Pimpaneau, Volker Schünemann, Antonio J. Pierik, Rolandas Meškys, Agota Aučynaitė, Matthias Boll

**Affiliations:** 1 Faculty of Biology – Microbiology, University of Freiburg, 79104 Freiburg, Germany; 2 Department of Molecular Microbiology and Biotechnology, Institute of Biochemistry, Life Sciences Center, Vilnius University, 10257 Vilnius, Lithuania; 3 Department of Physics, RPTU Kaiserslautern-Landau, 67663 Kaiserslautern, Germany; 4 Laboratoire de Chimie des Processus Biologiques, UMR 8229 CNRS, Collège de France, Sorbonne Université, Paris, CEDEX 05 France; 5 Department of Chemistry, RPTU Kaiserslautern-Landau, 67663 Kaiserslautern, Germany

**Keywords:** Transferases, RNA, Biocatalysis

## Abstract

In all domains of life, transfer RNAs (tRNAs) contain post-transcriptionally sulfur-modified nucleosides such as 2- and 4-thiouridine. We have previously reported that a recombinant [4Fe-4S] cluster-containing bacterial desulfidase (TudS) from an uncultured bacterium catalyzes the desulfuration of 2- and 4-thiouracil via a [4Fe-5S] cluster intermediate. However, the in vivo function of TudS enzymes has remained unclear and direct evidence for substrate binding to the [4Fe-4S] cluster during catalysis was lacking. Here, we provide kinetic evidence that 4-thiouridine-5’-monophosphate rather than sulfurated tRNA, thiouracil, thiouridine or 4-thiouridine-5’-triphosphate is the preferred substrate of TudS. The occurrence of sulfur- and substrate-bound catalytic intermediates was uncovered from the observed switch of the *S* = 3/2 spin state of the catalytic [4Fe-4S] cluster to a *S* = 1/2 spin state upon substrate addition. We show that a putative gene product from *Pseudomonas putida* KT2440 acts as a TudS desulfidase in vivo and conclude that TudS-like enzymes are widespread desulfidases involved in recycling and detoxifying tRNA-derived 4-thiouridine monophosphate nucleosides for RNA synthesis.

## Abstract (web summary)

TudS-like enzymes are desulfidases involved in recycling and detoxifying tRNA-derived 4-thiouridine monophosphate nucleosides for RNA synthesis as unveiled by enzymatic, spectroscopic and docking studies of two bacterial TudS enzymes.

## Introduction

Modified ribonucleic acids are present in all three domains of life, with more than 110 post-transcriptional modifications known for transfer RNAs (tRNAs)^1, 2^. Among them, sulfur modifications of tRNAs are essential for efficient and accurate translation and for the structural stability of tRNAs^3–6^. tRNAs are chemically modified after transcription of the tRNA genes and cleavage of the precursor tRNA^7^. Sulfur modifications are introduced by a sulfur metabolic network comprising cysteine desulfurases, sulfur carrier proteins, and sulfur transferases. Among the latter, an increasing number of FeS-dependent enzymes has been identified to be responsible for the thiolation of tRNA nucleotides^3,8–10^.

While the incorporation of sulfur into the tRNA has been intensively studied over the past decades, homeostasis and potential desulfurization of modified tRNAs and/or their degradation products have remained elusive^11–13^. Two tRNA degradation pathways for incompletely processed or misfolded tRNA are known in yeast^14^, but insights into the regulation and function of bacterial tRNA degradosomes, as a response to the presence of hypomodified tRNA or during oxidative stress, were reported only recently^15,16^. However, specific removal of post-transcriptional modifications of tRNA was reported only for enzymatic demethylation in the context of tRNA homeostasis^17–20^.

**Fig. 1:**
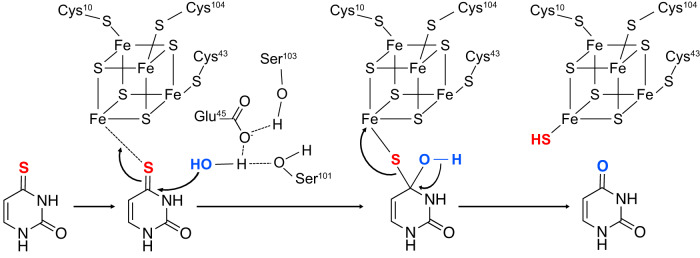
Proposed mechanism of TudS. The mechanism is based on the identification of a [4Fe-5S] intermediate in the crystal structure of TudS, modeling studies and site-directed mutagenesis^22^. Catalysis is assisted by Glu45, Ser101 and Ser103.

Recently, several gene products containing a Domain of Unknown Function 523 (DUF523) that produces uracil from 2-thiouracil were identified^21^. According to this activity, these proteins were designated TudS (thiouracil desulfidases). TudS from an uncultured bacterium, sharing the highest sequence identity (96%) to a TudS protein from a γ-proteobacterial *Aeromonas* species, was heterologously produced, its iron-sulfur cluster was reconstituted under anaerobic conditions, and the crystal structure of the holo-protein was solved^22^. The latter featured a [4Fe-4S]-cluster coordinated by three cysteines, leaving the fourth iron atom available for the coordination of exogenous ligands. The enzyme converted 4-thiouracil under anaerobic conditions at a higher rate than 2-thiouracil suggesting that the former represents the preferred substrate. Based on the structural identification of a substrate-induced [4Fe-5S] cluster along with modeling studies of the TudS-4-thiouracil complex and site-directed mutagenesis, a mechanism for desulfurization of the 4-thiouracil substrate was proposed. In this mechanism, catalysis is initiated by binding the substrate to the [4Fe-4S] cluster. The desulfurization reaction was performed by an activated water molecule attacking the C4 atom of the 4-thiouracil substrate, assisted by a conserved glutamate and two invariant serines, yielding uracil and the structurally characterized [4Fe-5S] intermediate (Fig. 1)^22^. These results revealed a novel function of the DUF523 protein family and deciphered a previously undescribed function of an iron-sulfur cluster as a sulfide acceptor in a desulfuration reaction.

The trapping of a [4Fe-5S] cluster intermediate in TudS upon incubation of the holo-TudS crystals in the presence of 4-thiouracil supported the proposed mechanism. However, no direct evidence for the binding of the substrate to the [4Fe-4S] cluster was obtained. Moreover, though the desulfurization of 2- and 4-thiouracil to uracil was demonstrated in vitro, the in vivo substrate of TudS and, consequently, its cellular function remained unclear as only the thiouracil bases were tested as potential substrates. There were two scenarios for the in vivo function: (i) TudS could be involved in the recycling of tRNA-derived sulfur-containing degradation products either on the level of the bases, nucleosides, or phosphate nucleosides. (ii) TudS might act on modified tRNA, which would suggest a regulatory role in tRNA function and/or stability.

Here, we show that TudS enzymes use 4-thiouridine-5’-monophosphate (4-thio-UMP) as the preferred substrate, strongly suggesting a role in the recycling of RNAse-mediated tRNA degradation products. No evidence for desulfurization of a synthetic 4-thiouridine-containing mini-tRNA was obtained. Further, we provide electron paramagnetic resonance (EPR) spectroscopic evidence for a marked switch of the spin state of the catalytic [4Fe-4S] cluster as a direct result of substrate binding.

## Results and discussion

### Heterologous production, purification and molecular properties of TudS enzymes

Though present in all domains of life, members of the TudS family (previously referred to as DUF523) are predominantly found in bacteria, mostly in proteobacteria and firmicutes. Here, we studied two TudS family members from an uncultured γ-proteobacterial bacterium sharing up to 96% sequence identity with that of an *Aeromonas* sp. that was also used in previous studies (AUF71791.1, referred to as TudS_A)^21^, and from *Pseudomonas* sp. MIL 19 (WP_274089503.1, referred to as TudS_P). The genes encoding TudS_A and TudS_P were heterologously expressed in uracil auxotrophic *Escherichia coli* cells cultured on a defined synthetic medium supplemented with 2- or 4-thiouracil. Notably, the genome of *E. coli* does not contain a gene encoding a putative TudS enzyme. Growth of both recombinant strains was observed in the presence of 2- and 4-thiouracil as well as 2- and 4-thiouridine indicating that the strains were capable of desulfurizing 2- and 4-thiouracil containing moieties in vivo (Supplementary Fig. S1).

TudS_A and TudS_P were produced anaerobically with a C-terminal His-tag in an *E. coli* expression strain lacking the Δ*iscR* gene. IscR is a repressor of the *isc* operon involved in the biogenesis of [Fe-S] clusters, and its deletion should promote the synthesis of [4Fe-4S] clusters^23^. Both proteins were purified by nickel (TudS_A) or cobalt (TudS_P) affinity chromatography, under either aerobic conditions followed by [4Fe-4S] cluster reconstitution as reported previously^22^, or under anaerobic conditions. The purity of anaerobically purified recombinant TudS proteins was analyzed by SDS-polyacrylamide gel electrophoresis (PAGE) (Supplementary Fig. S2A). On a molecular size exclusion chromatography column, TudS_A eluted as a 17.0 ± 1.5 kDa protein, indicating a monomeric architecture (theoretical molar mass of ≈17.6 kDa). The TudS_P protein eluted at 19.6 ± 1.5 kDa, also suggesting a monomer (theoretical mollar mass of ≈18.5 kDa, Supplementary Fig. S2B).

Previous studies succeeded in cluster reconstitution of TudS under anaerobic conditions using 100 µM apo-TudS_A, L-cysteine, Fe(II) and L-cysteine desulfurase, leading to 3.8 Fe ± 0.3 mol per monomer^22^. This value was in accordance with the [4Fe-4S] occupancy observed in the crystal structure. Using the same reconstitution system, we determined here a similar iron content for both TudS enzymes, ranging from 3.8 to 4.1 Fe per monomer. Both proteins exhibited an ultraviolet (UV)-visible spectrum characteristic of a [4Fe-4S]^2+^ cluster that was partially bleached upon reduction by dithionite (Supplementary Fig. S2C).

### The TudS-catalyzed reaction is independent of the redox state of the [4Fe-4S] cluster

Next, we investigated the effect of the redox state of the cluster on the catalytic activity and tested potential inhibitors of TudS enzymes using 4-thiouracil as a substrate. TudS_A activity was neither affected by reducing agents (Ti(III)-citrate, sodium dithionite, 5 mM each, Supplementary Fig S3A) nor oxidizing agents (thionine acetate, 2,6-dichlorophenolindophenol, potassium ferricyanide, methylene blue, 5 mM each, Supplementary Fig. S3A). This finding confirms that the non-redox reaction catalyzed by TudS desulfidases is independent of the redox state of the [4Fe-4S] cluster. We further tested the effect of sodium sulfide on TudS enzymes since a hydrogenosulfide ion was found bound to the fourth non-protein bonded Fe atom (Fe4) of the [4Fe-4S] cluster upon soaking crystals with 4-thiouracil^22^^,24^. Using 50–250 µM Na_2_S, no effect on the enzymatic activity of TudS_A was observed, indicating no inhibition (Supplementary Fig. S3B). Due to the structural similarity to 2-thiouracil and 4-thiouracil, cytosine and isocytosine were also tested as possible inhibitors, but both molecules had no effect on the catalytic activity of TudS_A (Supplementary Fig. S3C).

### TudS enzymes are 4-thio-UMP desulfidases

Though previous work identified a 2- and 4-thiouracil desulfurizing activity of TudS_A^22^, the natural substrate(s) and thus the cellular function of TudS enzymes remained an issue. Here, we measured the activity of TudS_A and TudS_P, produced and enriched anaerobically, with 2-thiouracil, 4-thiouracil, 2-thiouridine, 4-thiouridine, as well as 4-thio-UMP and 4-thio-UTP as possible substrates. Time-dependent substrate consumption and product formation were monitored by ultra-performance liquid chromatography (UPLC) coupled to photodiode array detection (Table 1). In agreement with previous observations^22^, both enzymes desulfurized 2-thiouracil and 4-thiouracil nearly completely at 0.5 mM substrate concentration, with the latter being converted 50- to 100-fold faster. When the substrate concentration was varied, the data obtained with 4-thiouracil fitted to a Michaelis–Menten curve, but not those obtained with 2-thiouracil (Supplementary Fig. S4). This result strongly indicates that TudS enzymes bind specifically only 4-thiouracil but not 2-thiouracil. When 2,4-dithiouracil was used as a substrate, the sulfur atom was abstracted first at the 4-position, with 2-thiouracil being formed as an intermediate that was further desulfurized into uracil (Supplementary Fig. S5).

**Table 1:** Catalytic parameters for desulfurization of 4-thiouracil, 4-thiouridine, 4-thio-UMP and 4-thio-UTP by TudS.

Enzyme	Substrate		*k*_cat_ (s^-1^)	*K*_m_ (mM)	*k*_cat_/*K*_m_ (M^-1^ s^-1^)	*V*_max_ (U mg^–1^)
TudS_A	4-thiouracil		0.70 ± 0.05	0.24 ± 0.03	2.90 × 10^3^	2.4 ± 0.2
TudS_A^R^			0.67 ± 0.06	0.7 ± 0.1	9.50 × 10^2^	2.3 ± 0.2
TudS_P			4.93 ± 0.43	1.49 ± 0.27	3.27 × 10^3^	15.9 ± 1.4
TudS_A	4-thiouridine		8.8 ± 0.9	3.1 ± 0.6	2.88 × 10^3^	30.1 ± 3.1
TudS_P			19.9 ± 4.7	0.2 ± 0.09	1.03 × 10^5^	64.3 ± 15.3
TudS_A	4-thiouridine monophosphate		293 ± 44	0.038 ± 0.013	7.77 × 10^6^	1000 ± 149
TudS_P			138 ± 16	0.020 ± 0.007	7.63 × 10^6^	447 ± 51
TudS_A	4-thiouridine triphosphate		68.6 ± 3.4	0.100 ± 0.023	6.85 × 10^5^	234 ± 12
TudS_P			52.3 ± 2.4	0.117 ± 0.026	4.48 × 10^5^	168 ± 8

Values are given for the anaerobically purified protein, except for TudS_A^R^, which is the protein with the reconstituted cluster. All values are normalized to 4 Fe per protein.

Next, we tested the possibility that thiouridine nucleosides and their mono- or triphosphate derivatives are desulfurized by the TudS enzymes (Table 1). For TudS_P, the *k*_cat_/*K*_m_ was >300-fold higher for 4-thiouridine than for 4-thiouracil, whereas for TudS_A, the increased *k*_cat_ for 4-thiouridine vs 4-thiouracil was accompanied by a higher *K*_m_. Similar to 2-thiouracil, the curve of the specific activity as a function of substrate concentration for 2-thiouridine showed no saturation when the substrate-dependence of the rate was analyzed (Supplementary Fig. S4F), again suggesting no specific binding of the 2-thio isomer. Strikingly, TudS_A and TudS_P exhibited the highest *k*_cat_/*K*_m_ values with 4-thio-UMP (2650- and 2230-fold higher than with 4-thiouracil, respectively), whereas for 4-thio-UTP, *k*_cat_/*K*_m_ values were around 10-fold lower than for 4-thio-UMP. These results indicate that 4-thio-UMP represents the preferred substrate for TudS enzymes, which however also desulfurize 4-thio-UTP formed from 4-thio-UMP in vivo, likely to prevent its incorporation into RNA.

**Fig. 2:**
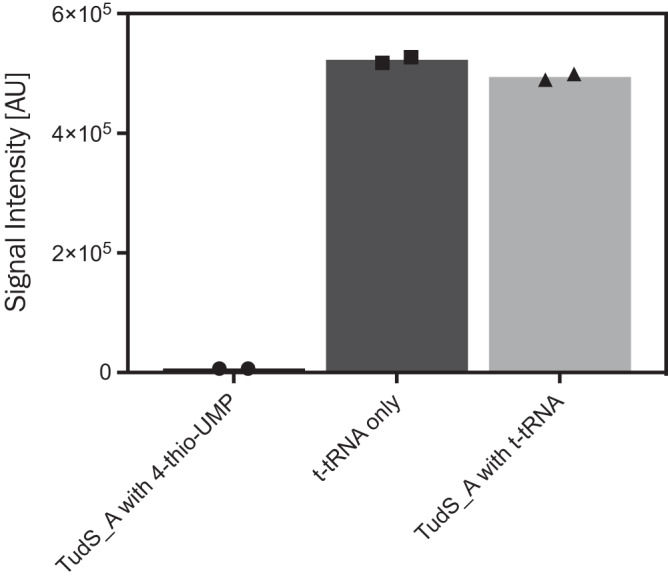
Reactivity of TudS_A with a 4-thiouridine-containing t-tRNA. TudS_A was reacted with a 4-thiouridine-containing t-tRNA after which samples were enzymatically hydrolyzed and subjected to UPLC-ESI-QTOF-MS analyses to monitor the conversion of the thiolated substrates. For each experiment, the 4-thiouracil fragments obtained after ESI ionization were analyzed quantitatively. Left column: positive control; middle column: negative control; right column: reaction mixture of TudS_A with t-tRNA.

We further tested the possibility that sulfurated tRNA could serve as a substrate for TudS enzymes. For this purpose, we tested a synthetic truncated tRNA (t-tRNA, Supplementary Fig. S6) harboring the conserved 4-thiouridine modification at position 8, which was designed according to previous reports^25,26^. Reaction mixtures containing t-tRNA alone (negative control), t-tRNA + TudS_A, or 4-thio-UMP + TudS_A (positive control) were incubated for 10 min at 30 °C followed by enzymatic hydrolysis to single nucleosides. The samples were analyzed by UPLC coupled to electrospray ionization quadrupole time-of-flight mass spectrometry (ESI-QTOF-MS). Notably, since the glycosidic bond of 4-thiouridine is cleaved during ESI ionization, the detection of 4-thiouracil was monitored^27^. Only traces of the 4-thiouracil moiety-containing substrate were found when 4-thio-UMP was incubated with TudS_A (positive control), indicating that it was processed into uracil. In contrast, t-tRNA incubated with or without TudS_A had a highly similar 4-thiouracil content (Fig. 2). These data suggest that the 4-thiouridine at position 8 of the t-tRNA used in this assay was not desulfurized by TudS_A.

Taking all kinetic data together, TudS enzymes preferentially act as 4-thio-UMP desulfidases but also display a catalytic activity with 4-thiouridine (1-2 orders of magnitude lower) and 4-thiouracil (2-3 orders of magnitude lower). This finding strongly suggests that the function of TudS is to recycle 4-thio-UMP that directly derives from hydrolytic cleavage of tRNAs by cellular RNAses^11^. Thus, our results showed no evidence for a role in regulating tRNA stability and function. The desulfurization of 4-thio-UMP is thought to be critical, not only for recycling tRNA-derived modified monophosphate nucleosides but also for preventing fatal erroneous incorporation of resulting 4-thio-uridine triphosphates into RNA during transcription^28^.

### Structural comparisons and computational docking studies provide a structural rationale for TudS substrate selectivity

**Fig. 3:**
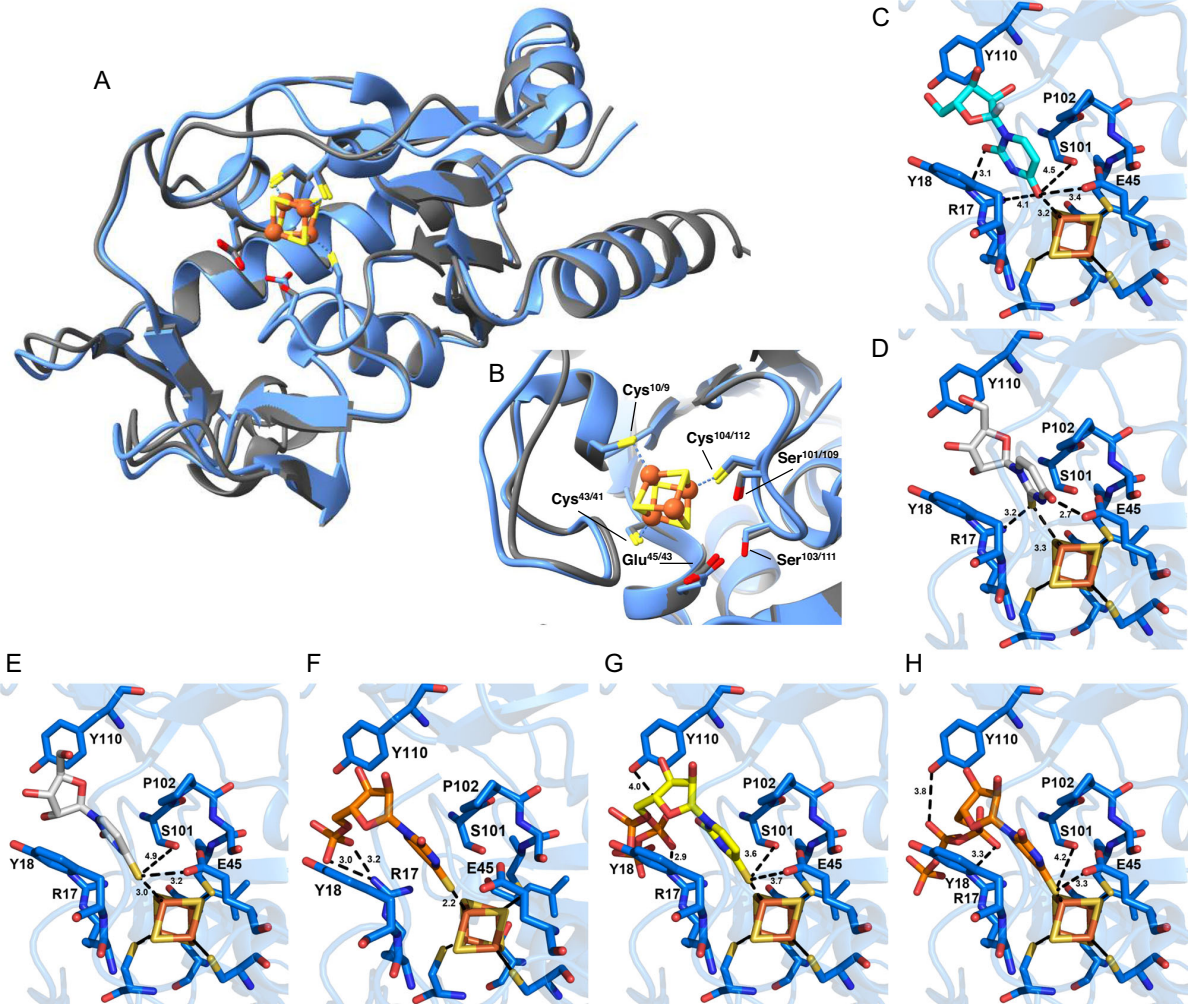
Comparison of TudS_A and TudS_P and docking models of TudS_A with various uracil derivatives. **A** Superposition of the TudS_P AlphaFold model (blue)^29^ onto the TudS_A crystal structure (gray)^22^ (PDB ID: 6Z96). **B** Close-up view of the active sites of the superposed models showing that the three cysteine residues (Cys10(TudS_A)/9(TudS_P), Cys43/41 and Cys104/112) that ligate the [4Fe-4S] cluster, as well as the conserved residues (Ser101/109, Ser103/111 and Glu45/43), which were proposed to assist in catalysis, occupy similar positions in the two proteins. Docking of uridine (**C**), 2-thiouridine (**D**), 4-thiouridine (**E**), 4-thio-UMP (**F**) and 4-thio-UTP (**G**, **H**) to TudS_A as calculated with Swissdock^31^. The distances between the ligand and selected TudS residues are given in Å.

A three-dimensional model of TudS_P (54% amino acid sequence identity with TudS_A) was generated using AlphaFold^29^ which was superimposed onto the TudS_A structure obtained at 1.8 Å resolution (PDB ID: 6Z96)^22^ using ChimeraX^30^ (Fig. 3). The model of TudS_P is compliant with the TudS_A crystal structure. In particular, the positions of the residues essential for catalysis are conserved in both TudS enzymes: the cysteines that ligate the [4Fe-4S]^2+^ cluster in TudS_A (Cys10, Cys43, Cys104) are equivalent to Cys9, Cys41, Cys112 in TudS_P, and the conserved glutamic acid and serine residues that were proposed to participate in catalysis TudS_A (Glu45, Ser101, Ser103) are equivalent to Glu43, Ser109, Ser111 in TudS_P (Fig. 3).

Previous docking studies showed that 2- and 4-thiouracil could be docked equally well to TudS using the EADock program, provided by the Swissdock web server^31^, according to the free energy of interaction^22^. However, the kinetic data obtained in this work indicate that 4-thiouridine nucleosides and their monophosphate derivatives are the preferred substrates of TudS enzymes. To provide a structural explanation for this observed selectivity, uridine, 2-, 4-thiouridine, 4-thio-UMP, and 4-thio-UTP were docked to the TudS_A structure using EADock_._ This program evaluates the protein-ligand binding energy using a scoring function based on the CHARMM22 force field^32^. For 4-thiouridine, 4-thio-UMP and 4-thio-UTP, the thio-group was found positioned close to the [4Fe-4S] cluster of TudS_A (Fig. 3, Supplementary Table S1). For uridine and 4-thiouridine, the cluster with the lowest energy corresponded to the conformation with the oxygen/sulfur positioned 3.0–3.3 Å away from the Fe4 atom. With 4-thio-UMP and 4-thio-UTP, this distance was even closer (2.2 Å), which is consistent with a covalent bond. Given the wide space sampling during the docking experiments, the short distance between the Fe4 atom and the sulfur atom of the substrate indicates a highly specific binding to TudS_A. In contrast to the docking of 4-thiouridine, constraints had to be applied to dock 2-thiouridine. In the lowest energy model, the thio group was 3.3 Å away from the Fe4 atom, with a binding energy of 0.5 kcal mol^–1^ above that for 4-thiouridine. This result indicates that 2-thiouridine is bound less specifically to TudS than 4-thiouridine, which is in full agreement with the kinetic data obtained.

The docking studies of uridine and 4-thiouridine derivatives indicate that the uracil base is sandwiched between Tyr18 and Pro102 and the sugar moiety between Tyr18 and Tyr110 (Fig. 3). Though the 4-thio-UMP and 4-thio-UTP docking results were similar to that of 4-thiouridine, additional specific ionic interactions between the conserved Arg17 of TudS_A and the phosphate group of the ligands were identified, which explain the preferred binding of 4-thio-UMP and 4-thio-UTP. In all models of the TudS_A complexes with thiouridine derivatives, the carbon atom linked to the sulfur atom is located in close proximity to Glu45 OE1 and Ser101 OG1 supporting their proposed role in catalysis (Fig. 1)^22^.

### EPR spectroscopy of TudS_A reveals direct binding of the substrate to the [4Fe-4S] cluster

The kinetic, structural and computational studies obtained so far suggest that the sulfur atom of 4-thiouracil-containing substrates forms a covalent bond with the ligand-free Fe4 atom of the [4Fe-4S] cluster during catalysis. To provide direct spectroscopic evidence for the existence of such an interaction, Mössbauer and electron paramagnetic resonance (EPR) spectroscopic studies were carried out using TudS_A as a model enzyme. For labeling with ^57^Fe and obtaining sufficiently high enzyme concentrations for the spectroscopic studies (400-500 µM), an upscaling of the production/reconstitution from the 1 mL to the >5 mL scale was necessary. However, this upscaling was generally accompanied by a decrease in iron content (to 3.1 ± 0.3 Fe mol per mol TudS_A).

The Mössbauer spectrum of TudS_A as isolated from recombinant *E. coli* cells grown in ^57^Fe-enriched medium (≥95%) showed an asymmetric doublet at 77 K that was assigned to a mixture of a [3Fe–4S]^+^ and a [4Fe–4S]^2+^ cluster (Supplementary Fig. S7). It could be simulated with a contribution of 79% [3Fe-4S]^+^ and 21% [4Fe-4S]^2+^. In the dithionite-reduced state, the ratio of the corresponding [3Fe-4S]^0^ and [4Fe-4S]^+^ did not change. Thus, the Mössbauer data are consistent with the decreased content in Fe observed when upscaling the reconstitution procedure for preparing the Mössbauer sample, and suggest a partial degradation of the [4Fe-4S] to the [3Fe-4S] cluster.

**Fig. 4:**
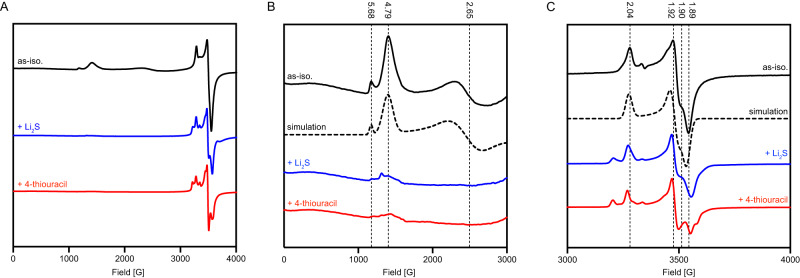
EPR spectra of dithionite-reduced TudS_A at different conditions. As isolated TudS_A without additions (black, simulations in dotted lines), with added 5 mM Li_2_S (blue), or with added 5 mM 4-thiouracil (red). **A** Broad magnetic field scan of TudS_A at 10 K. EPR conditions: microwave power, 209 mW; modulation amplitude, 1.5 mT; modulation frequency, 100 kHz. **B** Low-field region with *g*-values given for signals in samples without additions. **C** *g* ≈ 2 region. EPR conditions: microwave power, 0.00332 mW; modulation amplitude, 1.5 mT; modulation frequency, 100 kHz.

We investigated the effect of substrate binding to TudS_A by EPR spectroscopy. Oxidized [4Fe-4S]^2+^ and reduced [3Fe-4S]^0^ clusters are diamagnetic and not detectable by EPR. For this reason, EPR studies had to be conducted with reduced enzyme for analyzing the paramagnetic [4Fe-4S]^+^ cluster and with oxidized enzyme for [3Fe-4S]^+^ detection. Consistent with Mössbauer spectroscopic analysis, the as isolated enzyme (≈500 µM) exhibited only a signal typical for a [3Fe-4S]^+^ cluster in the *g* ≈ 2.0 region (Supplementary Fig. S8). Most importantly, addition of 4-thiouracil (5 mM) had only a very slight effect on this signal, which is in agreement with the proposed role of an intact [4Fe-4S] cluster as sulfide acceptor during TudS catalysis. Upon reduction of TudS_A with sodium dithionite (5 mM), in the absence of substrate, novel peaks in the *g* = 1.9 to 2.05 region were observed (Fig. 4A and C). These signals are typical of an *S* = 1/2 spin state of a [4Fe-4S]^+^ cluster, and could be simulated with *g*_xyz_-values of 2.04, 1.90, and 1.89. The *S* = 1/2 signal was accompanied by broad peaks in the low field region, with *g*-values at 5.68, 4.79 and 2.65 (Fig. 4B). The latter signal, typical for an *S* = 3/2 spin state of a [4Fe-4S]^+^ cluster, has been described for other [4Fe-4S] clusters, e.g., for the one in the Fe protein of nitrogenases^33,34^. Thus, similar to the nitrogenase Fe protein, the TudS_A [4Fe-4S]^+^ cluster was present in mixed populations of spin states with 45% *S* = 1/2 and 55% *S* = 3/2.

Upon addition of 4-thiouracil, marked changes were observed. Firstly, the *S* = 3/2 signal almost completely disappeared (Fig. 4B), accompanied by an appropriate increase of the *S* = 1/2 signal (Fig. 4C). An accurate quantification of the *S* = 1/2 signals in the samples with and without 4-thiouracil was hampered by the degree of reduction of the [4Fe-4S] cluster that varied from sample to sample but never reached 100%. Secondly, the *S* = 1/2 signal changed, with novel features appearing at *g* = 2.09 and *g* = 1.91 (Fig. 4C). To test whether these effects can be assigned to substrate- or sulfide-bound cluster species, we incubated reduced TudS_A with 5 mM Li_2_S. Similar to the effect produced by 4-thiouracil addition, the *S* = 3/2 signal fully disappeared upon Li_2_S addition (Fig. 4B). However, the *S* = 1/2 signal in the presence of lithium sulfide differed from the reduced TudS_A incubated with 4-thiouracil (Fig. 4C) suggesting that in the latter state the active site binds either the sulfide plus uracil products or 4-thiouracil. In particular, the features around *g* = 1.9 were more similar to the enzyme without any added substrate.

In summary, the Mössbauer and EPR spectroscopic analyses revealed that at high enzyme concentrations the cluster reconstitution of TudS_A is less effective than previously reported^22^ yielding a major fraction of enzyme harboring a [3Fe-4S] cluster. The switch between an active [4Fe-4S] cluster to an inactive [3Fe-4S] cluster upon loss of one Fe atom has initially been uncovered and intensively studied in aconitase^35^. To our advantage, the selective EPR detection of the enzyme fraction harboring a [4Fe-4S] cluster showed a very pronounced effect upon substrate binding, as indicated by a shift from a mixture of high- and low-spin states to a full low-spin state, accompanied by a marked change of the resulting *S* = 1/2 EPR signal. While the former effect was also observed upon sulfide binding, likely corresponding to the structurally characterized [4Fe-5S] cluster^22^, the resulting *S* = 1/2 signal differs between sulfide- and substrate-incubated TudS_A (Fig. 4C). The signal of reduced TudS_A after incubation with thiouracil is difficult to interpret and is probably due to a mixture of different low-spin *S* = 1/2 states of the [4Fe-4S]^+^ cluster that may be assigned either to a [4Fe-4S] cluster with 4-thiouracil ligated to the Fe4 atom, a [4Fe-5S] cluster with uracil bound to the enzyme or a mixture of both states. Anyhow, all interpretations are in favor of direct binding of 4-thiouracil to the Fe4 of the cluster with the observed EPR signals representing either the 4-thiouracil or the sulfide plus uracil bound state. Notably, the sulfide product did not act as an inhibitor of the TudS-catalyzed reaction, suggesting that it is readily substituted by the substrate during catalysis.

### TudS from *Pseudomonas putida* KT2440 enables growth with 4-thiouracil and 4-thiouridine as uracil/uridine sources but does not appear to be involved in tRNA desulfurization

**Fig. 5:**
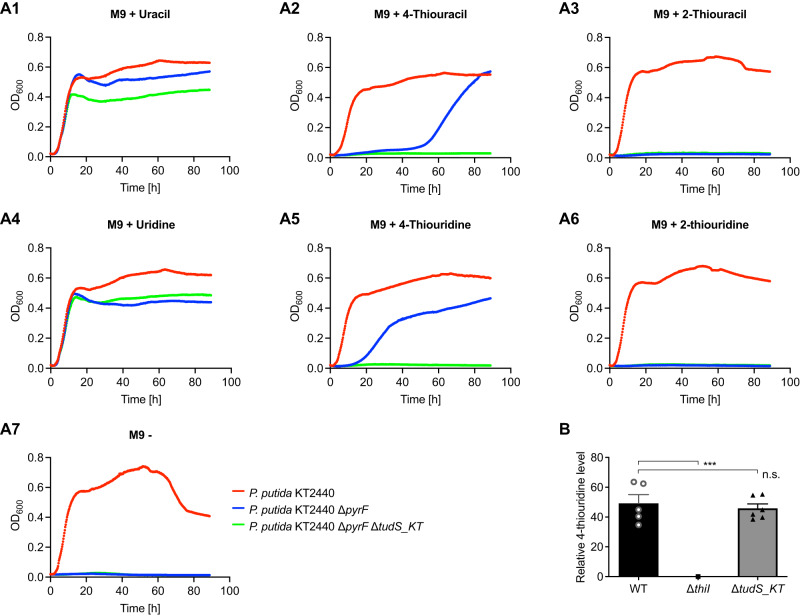
In vivo function of TudS_KT protein in *Pseudomonas putida* KT2440. **A** Growth curves of wild type, Δ*pyrF*, and Δ*pyrF*Δ*tudS_KT* knockout strains in the presence of (thio)uracil and (thio)uridine compounds (*n* = 3). M9 minimal media without any supplement was used as a negative control (A7, after 60 h cell lysis occurred) or was supplemented with 200 μM of nucleobases or nucleosides as indicated above (A1–A6). **B** 4-thiouridine level in tRNA from wild type (WT, *n* = 5, SE), from a tRNA sulfur transferase knockout strain (*ΔthiI*, *n* = 1), and from *ΔtudS_KT* knockout strain (*n* = 6, SE); 4-thiouridine was determined by mass spectrometry, and the relative levels determined were normalized to the total amount of cellular dihydrouridine^51^.

Among proteins of the DUF523 family, only the recombinant TudS_A and TudS_P were experimentally identified so far as 4-thio-UMP desulfidases. To further substantiate the function of TudS proteins, we studied native TudS from the genetically tractable *Pseudomonas putida* KT2440 (AAN70723.1), henceforth referred to as TudS_KT. An amino acid sequence alignment with TudS_A and TudS_P shows that the invariant residues involved in cluster binding and catalysis are present in TudS_KT (Supplementary Fig. S9). We created uracil auxotrophic Δ*pyrF* (necessary for de novo synthesis of uridine) and Δ*pyrFΔtudS_KT* knockout strains of *P. putida* KT2440, and investigated whether these strains were capable of proliferating in minimal medium containing 2- or 4-thiouracil containing compounds as sole sources of uracil (Fig. 5A). Growth of the single knockout Δ*pyrF* strain strictly depended on the presence of uracil (Fig. 5 A1) or uridine (Fig. 5 A4), and could also be recovered with 4-thiouracil/4-thiouridine. However, growth with the latter two was only observed after a significant lag phase, which may be explained by gene induction and de novo synthesis of TudS_KT and other enzymes (e.g., kinases for UTP synthesis) (Fig. 5, A2, A5). In contrast, no growth was observed with 2-thiouracil/2-thiouridine (Fig. 5, A3, A6), which is different to the *E. coli* HMS174Δ*pyrF* mutant (TudS_A producing) that grew with 2-thiouracil (Supplementary Fig. S1). This result indicates that the endogenous TudS_KT has a clear preference for 4-thiouracil over 2-thiouracil moiety-containing compounds, which is in line with our in vitro studies with recombinant TudS_A and TudS_P. As a control, the double knock-out Δ*pyrFΔtudS_KT* strain grew only in the presence of uracil (Fig. 5, A1)/uridine (Fig. 5 A4) but not in the presence of 4-thiouracil moiety-containing compounds (Fig. 5, A2, A5), confirming that TudS_KT enables the use of 4-thiouracil/4-thiouridine as exogenous uracil/uridine sources.

Next, we tested the effect of the *ΔtudS_KT* knockout on the 4-thiouridine content of tRNA from *P. putida* KT2440 (Fig. 5B). For this purpose, we isolated bulk tRNA from the wild type, the *ΔtudS_KT* knockout, and from a *ΔthiI* knockout, in which a gene encoding the tRNA sulfur transferase involved in 4-thiouridine formation^9,36^ is deleted, and the 4-thiouridine content of each strain was determined by mass spectrometry. As expected, the content of 4-thiouridine in bulk tRNA was drastically reduced in the *ΔthiI* knockout strain (negative control). However, virtually no difference was observed between the wild type and the *ΔtudS_KT* strains. This result shows that TudS_KT does not act on 4-thiouridines incorporated in tRNA in vivo, indicating that it is rather involved in recycling 4-thiouracil, 4-thiouridine, and 4-thio-UMP.

## Conclusions

In this work, we provide experimental evidence that TudS enzymes of the DUF523 family, present in bacteria, archaea and also some eukaryotes, are desulfidases acting on thiolated bases, nucleosides and their monophosphate and triphosphate derivatives with a preference for 4-thio-UMP. Indeed, the TudS_A and TudS_P enzymes both showed the highest *k*_cat_/*K*_m_ values for 4-thio-UMP, followed by 4-thio-UTP and 4-thiouridine, whereas kinetic studies indicated no specific binding of 2-thiouracil/2-thiouridine. These results are in full agreement with docking studies that showed the best energy of interaction with 4-thio-UMP and 4-thio-UTP, with an arginine making an ionic interaction with the phosphate group of the substrate, and a distance between the Fe4 atom of the [4Fe-4S] cluster and the sulfur atom of the substrate corresponding to a covalent bond. In contrast, no evidence for a function of TudS as a thiolated tRNA desulfidase was obtained, neither in vitro using a synthetic t-tRNA nor in vivo in *P. putida* KT2440. However, desulfuration of full-length tRNAs by TudS enzymes in other organisms cannot be ruled out by this study. Notably, TudS enzymes are often fused to additional domains, most frequently to the DUF1722 domain^21^, which may mediate an altered substrate preference with regard to the accommodation of larger substrates such as oligonucleotides or even tRNA.

The substrate preference for 4-thio-UMP suggests that TudS enzymes are involved in recycling monophosphate nucleosides, the primary RNAse-mediated tRNA degradation products, rather than the corresponding nucleosides or bases. However, the even more important cellular function of TudS proteins is likely to convert the thiolated monophosphate nucleosides into standard building blocks for new RNA synthesis. This function is important to avoid the formation of thiolated triphosphate nucleosides by cellular uridylate and nucleoside diphosphate kinases and their consecutive integration during the transcription of RNA species. Such a toxic effect of 4-thiouridine incorporation has been reported for elevated thiouracil concentration (50 µM) that inhibited the production and processing of 47S rRNA, triggering a nucleolar stress response^28^.

After the recent identification of the [4Fe-5S] cluster species upon soaking TudS crystals with 4-thiouracil^22^, the present work strongly suggests that TudS catalysis involves the direct binding of the 4-thiouracil-containing substrates to the [4Fe-4S] cluster. The changes of the EPR spectroscopic properties, induced by 4-thiouracil binding, are remarkable and indicate strong effects on the spin coupling within the [4Fe-4S] core upon substrate binding to the cluster. It is noteworthy that sulfur transferases acting on nucleosides employ catalytic [4Fe-4S] clusters and similar mechanisms for sulfuration and desulfuration^24^, although tRNA sulfur transferases and TudS-like desulfidases are phylogenetically not related.

## Materials and methods

### Bacterial strains, plasmids, and oligonucleotide primers

The bacterial strains used in this study are listed in Supplementary Table S2. The plasmid vectors used in this study are listed in Supplementary Table S3. The oligonucleotide primers used in this study are listed in Supplementary Table S4. Standard techniques were used for DNA manipulations^37^. DNA primers were synthesized by Metabion International AG. DNA sequencing was performed by Azenta, Germany.

### In vivo activity of recombinant TudS proteins

The in vivo activity of TudS_A (AUF71791.1) and TudS_P (WP_274089503.1) proteins was tested using the uracil auxotrophic *E. coli* HMS174Δ*pyrF* cells^22^, carrying the TudS_A and TudS_P encoding plasmids pLATE11-tudS_A and pLATE11-*DUF523_PP_A* respectively. Cells were grown aerobically in LB medium supplemented with 100 μg/mL ampicillin at 37 °C until OD_600_ reached 1. Serial dilutions were performed in sterile 0.9% NaCl solution. The diluted cell samples were grown aerobically at 37 °C on agar M9 minimal medium supplemented with 100 μg/mL ampicillin, 0.1 mM IPTG, and 20 μg mL^–1^ or 40 µg mL^–1^ (thio)uracils or (thio)uridines as sources of uracil.

### Heterologous *TudS* gene expression in *E. coli*

Anaerobic expression of the genes encoding the TudS_A and TudS_P proteins was carried out in *E. coli* BL21(DE-3)Δ*iscR*^23^ strain. Bacterial cells were grown in TB medium (89 mM KH_2_PO_4_/K_2_HPO_4_ at pH 7.5, 12 g/L tryptone, 24 g/L yeast extract, 0.4% (TudS_A)/0.2% (TudS_P) (v/v) glycerol, 50 mM fumaric acid, 200 µM (TudS_A)/760 μM (TudS_P) Fe(III)-citrate, 100 μg mL^–1^ ampicillin, 75 μg mL^–1^ kanamycin). After inoculation, the culture bottles were sealed with rubber stoppers, and air in the headspace was flushed with nitrogen. Cells were incubated at 37 °C until OD_600_ reached 0.4–0.7. Gene expression was induced with 0.5 (TudS_A)/0.75 mM (TudS_P) IPTG, and growth medium was supplemented with 1 mL/L vitamin solution VL-7^38^, trace element solution SL-9^39^, 0.1 mM CaCl_2_, 0.8 mM MgSO_4_ and 3 mM NaNO_3_. Cells were incubated for 20 h at 16 °C (TudS_A)/20 °C (TudS_P) with agitation of 100 rpm. Cells were harvested anaerobically by centrifugation at 4500 × *g* (4 °C) for 20 min and used directly or stored at −80 °C in anaerobically sealed bottles.

### Anaerobic enrichment of recombinant proteins

The cell lysate was centrifuged for 1 h at 150,000 × *g*. The supernatant was filtered with a 0.2-µm filter, and the recombinant TudS protein was enriched using an Äkta pure FPLC system (Cytiva). TudS was purified on 5 mL HisTrap™ HP His-Tag protein purification columns (TudS_A, Cytiva) or 5 mL HiTrap TALON crude resins (TudS_P, Cytiva) previously equilibrated with buffer A (TudS_A: 20 mM HEPES pH 7.5, 150 mM NaCl, 5% (v/v) glycerol, ∼0.1 mg L^–1^ DNase I, 5 mM imidazole; TudS_P: 20 mM HEPES pH 7.5, 300 mM NaCl, ∼0.1 mg L^–1^ DNase I, ∼0.1 mg L^–1^ RNase A, ∼0.1 mg L^–1^ lysozyme, 10 mM imidazole). After washing the column with 2 column volumes of 50 mM imidazole (TudS_A) or 10 column volumes of buffer A (TudS_P), the adsorbed protein was eluted with 200 mM imidazole (TudS_A) or a linear gradient of 10–400 mM imidazole over 10 column volumes (TudS_P). Proteins were concentrated to 10–35 mg/mL using Vivaspin™ Turbo 15 RC 10000 MWCO concentrators (Sartorius). The buffer was adjusted to 20 mM HEPES pH 7.3, 150 mM NaCl (TudS_P: 300 mM), and 5 % (v/v) glycerol using PD-10 or G-25 desalting columns (Cytiva).

Enriched enzymes were stored in anaerobically sealed tubes at −80 °C. The purity of the recombinant proteins was confirmed by electrophoresis on 12.5–16% SDS-PAGE gels. The concentration of recombinant proteins was measured by the Bradford^40^ method with bovine serum albumin as the calibration standard. The iron content was determined as described previously^41^ using the method of Lovenberg et al.^42^ and the Ferene test^43^.

### Determination of TudS molar mass

The molar masses of TudS_A and TudS_P were determined by analytical gel filtration using a Superdex 75 10/300 GL column (Cytiva) previously equilibrated with 20 mM HEPES pH 7.5, 150 mM NaCl, 5% (w/v) glycerol, under anaerobic conditions. Bovine serum albumin (67 kDa), carbonic anhydrase (29 kDa) and cytochrome c (12,4 kDa) were used as standards for column calibration. The experiment was repeated three times to determine the standard deviation.

### UV-visible spectroscopy

UV-visible spectra of purified recombinant TudS enzymes were recorded on a Shimadzu UV-1800 Spectrophotometer in a quartz cuvette under strictly anaerobic conditions.

### Enzyme activity measurements

Enzymatic activity tests of TudS_A and TudS_P were carried out under anaerobic conditions in a glove box (95% N_2_, 5% H_2_). The activity of TudS_A was analyzed by analyzing the reaction mixtures by Ultra performance liquid chromatography (UPLC). The various substrates (0–5000 µM), 4-thiouracil (Sigma-Aldrich, Inc.), 2-thiouracil (Sigma-Aldrich, Inc.), 4-thiouridine (Jena Bioscience GmbH), 2-thiouridine (Jena Bioscience GmbH), 4-thio-UMP (Jena Bioscience GmbH) and 4-thio-UTP (Jena Bioscience GmbH) were incubated with recombinant TudS_A (0–25 µM) at 30°C for 30 s in 40-50 μL of 100 mM MOPS/KOH pH 7.3. Na_2_S (0–250 µM) and cytosine or isocytosine (0.25–1 mM) were tested as inhibitors of TudS_A. Redox agents (titanium-(III)-citrate, thionine acetate, sodium dithionite, 2,6-dichlorophenolindophenol, methylene blue, potassium ferricyanide) were added a concentration of 5 mM to test their effect on the enzymatic activity. Reactions were started by addition of the enzyme and stopped by adding 5 μL of formic acid (2-thiouracil, 2,4-dithiouracil, 4-thiouracil, partly 4-thiouridine), heating the samples to 95 °C (4-thiouridine) or addition of 5 µL of sodium dodecyl sulfate (10% (w/v), 4-thio-UTP). The proteins were precipitated and supernatants were analyzed on an Acquity H-Class UPLC (Waters) by reversed-phase liquid chromatography (Waters Acquity UPLC HSS T3 2.1×100 mm column, 1.8 µm particle size) using a gradient of water/acetonitrile containing 0.1% formic acid. The identity and concentrations of the substrates and products were determined from their retention times and spectra by comparison with standards. For TudS_A and TudS_P, conversion of low substrate concentrations of <150 µM (4-thio-UMP and 4-thio-UTP, for TudS_P also 4-thiouracil and 4-thiouridine) was assayed using a continuous spectrophotometric assay measuring the specific decrease in substrate absorbance (extinction coefficients provided by the manufacturers).

The TudS_P activity towards substrates (4-thiouracil, 4-thiouridine, 4-thio-UTP) assayed at a concentration of 0.25–3.75 mM was measured by UPLC. The assays were performed in 40–50 µl of 100 mM MOPS/KOH pH 7.3 and started by adding 0.02–5 µM of recombinant TudS_P. The catalytic constants were determined using the Prism software package (GraphPad) by fitting the reaction rates obtained at different substrate concentrations to Michaelis–Menten curves.

### TudS_A activity measurement with t-tRNA

*Aeromonas hydrophila* tRNA_Ala_ (tRNAdb ID^44^: tdbD00000071) was used as a template to create a t-tRNA, consisting of 39 nucleotides (Supplementary Fig. S6, 5’-GGGGCUAS^4^UAGGGUCUGCGGGAAACCGCAUAGCUCCACCA-3’). The truncated t-tRNA, consisting of the acceptor stem, the s^4^U modification at position 8, a shortened T-loop and variable region of the template tRNA, was synthesized by Horizon Discovery Ltd. The t-tRNA (200 µM) was incubated with TudS_A (72 µM) at 30 °C under anaerobic conditions for 10 min in 100 mM MOPS/KOH at pH 7.3 (50 µL), stopped at 95 °C for 2 min and centrifuged at 14,000 rpm at 4 °C for 20 min. 10 μL of the supernatant were treated with Benzonase, Phosphodiesterase I and Alkaline Phosphatase at 37 °C for 3 h and filtered (10 kDa)^45^. 5 μL of the flow-through was separated by reversed-phase liquid chromatography (Acquity HSS T3 2.1 × 100 mm resin, 1.8 µm particle size, Acquity I Class UPLC, Waters) and analyzed by UV-visible spectroscopy (Acquity UPLC, Waters) and ESI/Q-TOF-MS (G2-Si, Waters).

### *Pseudomonas putida* mutant strains

*P. putida* mutant strains (Supplementary Table S2) bearing single or double marker-less gene disruptions were obtained by double crossover recombination technique according to the modified protocol by Oh et al.^46^. Two ~500 bp regions upstream and downstream of the gene of interest were amplified by PCR (primers are listed in Supplementary Table S4) directly from *P. putida* suspension in water. A kanamycin resistance cassette was amplified from FRT-PGK-gb2-neo-FRT PCR-template (Gene Bridges, Germany). Two genomic DNA fragments and a kanamycin resistance cassette were joined by overlap PCR and the product was cloned into *Sma*I-digested pUC19_sacB suicide vector^47^. *P. putida* cells were transformed by electroporation^48^. Kanamycin-resistant crossovers were selected on LB agar plates supplemented with 100 µg ml^–1^ kanamycin, grown at 30 °C overnight. The first crossover was confirmed by streaking colonies on LB agar plate containing 20% sucrose, grown at 30 °C overnight. The positive clones demonstrated characteristic blurred shape and slower growth. A single sucrose-sensible clone was grown for 6 h in LB media at 37 °C and serial dilutions were plated on LB agar plate containing 1 mg mL^–1^ 5-fluoroorotic acid (FOA) or 20% sucrose to detect *pyrF or thiI* and *tudS_KT* knockouts, respectively. Sucrose or FOA-resistant, kanamycin-sensitive second crossovers were confirmed by PCR using specific primers covering a region upstream and downstream knocked out gene. The final *ΔpyrF*, *ΔthiI*, *ΔtudS_KT*, and *ΔpyrFΔtudS_KT* marker-less mutations were confirmed by cloning the region of interest into pUC19 vector and sequencing. To obtain the *Pseudomonas putida* KT2440 growth curves the wild-type bacteria were grown in liquid LB medium, the *pyrF* knock-out strains were grown in liquid LB medium supplemented with 1 mM uracil overnight at 37 °C. The resulting cultures were washed from uracil once using 0.9% NaCl, resuspended, and liquid M9 minimal media (1× M9 salts, 1 mM MgSO_4_, 0.05 mM CaCl_2_, 0.2% glucose) supplemented with nucleobases and nucleosides (200 µM final concentration) was innoculated uniformly to OD_600_ 0.02. Bacterial growth was monitored using Tecan Infinite M200 PRO microplate reader, cultures were grown in 96 well flat bottom plates at 37 °C with periodic shaking every 5 min and OD_600_ measurements every 15 min.

### Preparation of bulk tRNA

Bulk tRNA was prepared as described in ref. ^49^ with slight modifications. 50 mL of bacterial culture was resuspended in extraction buffer followed by treatment with ROTI Aqua-Phenol for RNA extraction (ROTH, Germany) and the tRNAs in the aqueous phase were fractioned by ethanol precipitation. Solution of isolated tRNAs was loaded on HiTrap DEAE Sepharose FF column (Cytiva) and washed using FPLC system. Collected fractions were precipitated using ethanol and the pellet was resuspended in DEPC-treated water.

### Analysis of tRNA nucleoside composition

Prior to the reaction, tRNA samples were heat-denatured at 95 °C for 5 min. Enzymatic reaction (100 µL final volume) contained: 100 µg of bulk tRNA, 50 mM Tris-HCl pH 8, 10 mM MgCl_2_, 2U FastAP alkaline phosphatase (Thermo Fisher Scientific), 250U Pierce Universal Nuclease (Thermo Fisher Scientific) and 0.005U Phosphodiesterase I from *Crotalus atrox* (Sigma). Samples were incubated at 37 °C for 3 hours. The reaction was stopped and proteins in the reaction mixture were precipitated by adding 100 µL of acetonitrile and incubating at 37 °C, 1200 rpm for 10 min. The proteins were precipitated by centrifugation and supernatants were analyzed using liquid chromatography-tandem mass spectrometry on a Nexera X2 UHPLC system coupled with LCMS-8050 mass spectrometer (Shimadzu) equipped with an ESI source. The chromatographic separation was carried out using a 3 × 150 mm YMC-Triart C18 column (YMC) and a mobile phase that consisted of 0.1% formic acid and acetonitrile delivered in gradient elution mode. Modified nucleosides were detected using transitions *m/z* 247 → 115 (dihydrouridine) and 259 → 216 (4-thiouridine). The amount of 4-thiouridine was normalized to the total dihydrouridine content^50^.

### Modeling studies

Docking of the (thio)uridine derivatives was carried out with EADock, which is provided on the SwissDock web server^31^. Missing hydrogens in the TudS_A crystal structure were added using the “Structure editing/AddH” tool in UCSF CHIMERA^30^. The program was used in the default fast protocol except for 2-thiouridine, for which it was necessary to choose the “accurate” mode and local docking near the Fe4 atom of the cluster (10 × 10 × 10 Å^3^ box), with flexibility for amino acids within 3 Å of the ligand to get a binding mode with the sulfur atom of 2-thiouridine near the cluster. The clusters were classified according to the full fitness energy (Supplementary Table S1) but often the clusters with the lowest interaction energy gave more reasonable solutions (Fig. 3).

### Mössbauer spectroscopy

Transmission Mössbauer spectra were recorded with a conventional Mössbauer spectrometer operated in the constant acceleration mode in conjunction with a multi-channel analyzer in the timescale mode (WissEl GmbH). The spectrometer was calibrated against α-iron at room temperature. A flow cryostat (OptistatDN, Oxford Instruments) was used to measure the samples at 77 K. The spectral data were transferred from the multi-channel analyzer to a PC for further analysis employing the public domain program Vinda^51^ running on an Excel 2003® platform. The spectra were analyzed by least-squares fits using Lorentzian line shapes with the linewidth Γ. Samples of ^57^Fe-labeled TudS_A were prepared anaerobically at a concentration of 400 µM in 100 mM MOPS/KOH at pH 7.3, as isolated or with 15 mM 4-thiouridine.

### EPR spectroscopy

EPR spectroscopy employed an upgraded Elexsys E580 Bruker X-band spectrometer with 4122HQE resonator. With an Oxford Instruments ESR 900 helium continuous-flow cryostat the temperature of the samples was maintained at 10 K by cooling with a Stinger closed-cycle cryostat (Cold Edge Technologies) and an F-70 Sumitomo helium compressor. Simulations of EPR spectra were performed with easyspin^52^. Samples of 560–600 µM TudS_A in 100 mM [(tris(hydroxymethyl)methylamino]propanesulfonic acid (TAPS)/KOH pH 8.9 were reduced by incubation with 5 mM sodium dithionite and oxidized by incubation with 1 mM potassium ferricyanide. Li_2_S (150 mM) and 4-thiouracil (150 mM in DMSO) were added at a final concentration of 5 mM.

### Reporting summary

Further information on research design is available in the Nature Portfolio Reporting Summary linked to this article.

### Supplementary information

Supplemental Information

Description of Additional Supplementary Data

Supplementary Data 1

Reporting Summary

## Data Availability

Source data for all figures in the article and the supplementary figures are provided with this paper in the Supplementary Data items. The models of the various uridine compounds docked to the TudS crystal structure (PDB code 6Z96) are available with the following doi numbers at https://www.doi.org: TudS, 10.13140/RG.2.2.24594.63689; uridine, 10.13140/RG.2.2.27950.08005; 2-thiouridine 10.13140/RG.2.2.34660.96642; 4-thiouridine, 10.13140/RG.2.2.19561.47201; 4-thio-UMP, 10.13140/RG.2.2.32983.24483; 4-thio-UTP (cluster 2), 10.13140/RG.2.2.29627.80165; 4-thio-UTP (cluster 6), 10.13140/RG.2.2.24175.20645. Further data supporting this study are available from the corresponding author upon reasonable request.
