# Publisher Correction: TudS desulfidases recycle 4-thiouridine-5’-monophosphate at a catalytic [4Fe-4S] cluster

**DOI:** 10.1038/s42003-023-05625-0

**Published:** 2023-12-11

**Authors:** Jonathan Fuchs, Rapolas Jamontas, Maren Hellen Hoock, Jonathan Oltmanns, Béatrice Golinelli-Pimpaneau, Volker Schünemann, Antonio J. Pierik, Rolandas Meškys, Agota Aučynaitė, Matthias Boll

**Affiliations:** 1 Faculty of Biology – Microbiology, University of Freiburg, 79104 Freiburg, Germany; 2 Department of Molecular Microbiology and Biotechnology, Institute of Biochemistry, Life Sciences Center, Vilnius University, 10257 Vilnius, Lithuania; 3 Department of Physics, RPTU Kaiserslautern-Landau, 67663 Kaiserslautern, Germany; 4 Laboratoire de Chimie des Processus Biologiques, UMR 8229 CNRS, Collège de France, Sorbonne Université, Paris, CEDEX 05 France; 5 Department of Chemistry, RPTU Kaiserslautern-Landau, 67663 Kaiserslautern, Germany

**Keywords:** Transferases, RNA, Biocatalysis

Correction to: *Communications Biology* 10.1038/s42003-023-05450-5, published online 27 October 2023

In this article, the wrong structure for 4-thiouridine appeared in Table 1 in the PDF version of the article; the table should have appeared as shown below. The original article has been corrected.

**Table 1:** Catalytic parameters for desulfurization of 4-thiouracil, 4-thiouridine, 4-thio-UMP and 4-thio-UTP by TudS.

Enzyme	Substrate		*k*_cat_ (s^-1^)	*K*_m_ (mM)	*k*_cat_/*K*_m_ (M^-1^ s^-1^)	*V*_max_ (U mg^–1^)
TudS_A	4-thiouracil		0.70 ± 0.05	0.24 ± 0.03	2.90 × 10^3^	2.4 ± 0.2
TudS_A^R^			0.67 ± 0.06	0.7 ± 0.1	9.50 × 10^2^	2.3 ± 0.2
TudS_P			4.93 ± 0.43	1.49 ± 0.27	3.27 × 10^3^	15.9 ± 1.4
TudS_A	4-thiouridine		8.8 ± 0.9	3.1 ± 0.6	2.88 × 10^3^	30.1 ± 3.1
TudS_P			19.9 ± 4.7	0.2 ± 0.09	1.03 × 10^5^	64.3 ± 15.3
TudS_A	4-thiouridine monophosphate		293 ± 44	0.038 ± 0.013	7.77 × 10^6^	1000 ± 149
TudS_P			138 ± 16	0.020 ± 0.007	7.63 × 10^6^	447 ± 51
TudS_A	4-thiouridine triphosphate		68.6 ± 3.4	0.100 ± 0.023	6.85 × 10^5^	234 ± 12
TudS_P			52.3 ± 2.4	0.117 ± 0.026	4.48 × 10^5^	168 ± 8

Values are given for the anaerobically purified protein, except for TudS_A^R^, which is the protein with the reconstituted cluster. All values are normalized to 4 Fe per protein.

