# Supplementary material for: TudS desulfidases recycle 4-thiouridine-5’-monophosphate at a catalytic [4Fe-4S] cluster
Source: Commun Biol. 2023 Oct 27;6:1092. doi: 10.1038/s42003-023-05450-5 (PMC10611767; doi:10.1038/s42003-023-05450-5)
Supplement: Supplementary file 1 — Supplemental Information [file 42003_2023_5450_MOESM1_ESM.pdf]

# TudS desulfidases recycle 4-thiouridine-5´-monophosphate at a catalytic [4Fe-4S] cluster

Jonathan Fuchs[1^], Rapolas Jamontas[2^], Maren Hellen Hoock[3], Jonathan Oltmanns[3], Béatrice Golinelli-Pimpaneau[4], Volker Schünemann[3], Antonio Pierik[5], Rolandas Meškys[2], Agota Aučynaitė[2], Matthias Boll[1*]

## Supplementary information

**Supplementary Tables S1-S4**
**Supplementary Figures S1-S9**
**Supplementary References**

**Table S1. Parameters for the molecular docking scores of uridine derivatives.**

| Ligand | FullFitness (kcal/mol)[a] | Estimated ΔG[b] (kcal/mol) | d C(S)[c]-E45 OE1 (Å) | d C(S)- S101 O1G (Å) |
|---|---|---|---|---|
| Uridine (cluster 0) | -749.56 | -7.37 | | |
| 2-thiouridine (cluster 4) | -722.43 | -7.05 | 4.1 | 4.4 |
| 4-thiouridine (cluster 4) | -731.85 | -7.55 | 4.3 | 4.7 |
| 4-thio-UMP (cluster 6, conf. 3) | -876.49 | -9.67 | 4.0 | 3.8 |
| 4-thio-UTP (cluster 2, conf. 1) | -1187.84 | -18.48 | 4.0 | 3.9 |
| 4-thio-UTP (cluster 6, conf. 2) | -1182.422 | -17.54 | 3.4 | 4.0 |

[a] the FullFitness of a cluster is calculated by averaging the 30% most favorable effective energies of its elements to limit the risk of a few complexes penalizing the whole cluster. This effective energy is the sum of the total energy of the system and a solvation term.

[b] interaction energy, as calculated by EADock

[c] d C (S) designs the distance from the carbon atom that bears the sulfur atom: C2 or C4

"conf." stands for "conformation"

**Table S2. Bacterial strains used in this study.**

| | Comments | Source |
|---|---|---|
| *Escherichia coli* DH5Δα | Used for routine DNA manipulations | Pharmacia, USA |
| *Escherichia coli* BL21(DE3) | | Avidis, France |
| *Escherichia coli* BL21(DE3) Δ*iscR* | | [1] |
| *Escherichia coli* HMS174(DE3) Δ*pyrF* | | [2] |
| *Pseudomonas* sp. MIL19 | NCBI accession ID: PRJNA877084 | Isolated from soil; Vilnius university Life Sciences Center |
| *Pseudomonas putida* KT2440 | DSM No.: 6125 | DSMZ-German Collection of Microorganisms and Cell Cultures GmbH |
| *Pseudomonas putida* KT2440 Δ*tudS_KT* | Gene NCBI locus tag: PP_5158 | This study |
| *Pseudomonas putida* KT2440 Δ*thiI* | Gene NCBI locus tag: PP_5045 | This study |
| *Pseudomonas putida* KT2440 Δ*pyrF* | Gene NCBI locus tag: PP_1815 | This study |
| *Pseudomonas putida* KT2440 Δ*pyrF*Δ*tudS_KT* | | This study |

**Table S3. Plasmid vectors used in this study.**

| | Purpose | Source |
|---|---|---|
| pET21b | Expression vector for C-terminally 6xHis-tagged proteins | Novagen, Germany |
| pUC19_sacB | Suicide vector used for *P. putida* mutagenesis | [3] |
| pUC19 | Cloning vector | Thermo Fisher Scientific, USA |
| pET21b-DUF523_Vcz | Expression vector for recombinant TudS_A protein | [2] |
| pLATE11-DUF523_PP_A | Gene was amplified using PP_A_11_31_FW and PP_A_11_52_RV primers and cloned using aLICator LIC Cloning and Expression System (Thermo Fisher Scientific, USA) | This study |
| pLATE31-DUF523_PP_A | Gene was amplified using PP_A_11_31_FW and PP_A_31_RV primers and cloned using aLICator LIC Cloning and Expression System (Thermo Fisher Scientific, USA) | This study |
| pUC19_SacB_DUF523_mut | Suicide mutagenesis vector coding for *tudS* flanking region and a kanamycin casette. Upstream region was amplified using KT2440_DUF_1_FW and KT2440_DUF_2_RV primers, downstream region was amplified using KT2440_DUF_3_FW and KT2440_DUF_4_RV primers, kanamycin casette was amplified using FRT-PGK-gb2-neo-FRT_RV and FRT-PGK-gb2-neo-FRT_FW primers. | This study |
| pUC19_SacB_pyrF_mut | Suicide mutagenesis vector coding for *pyrF* flanking region and a kanamycin casette. Upstream region was amplified using KT2440_pyrF_1_FW and KT2440_ pyrF _2_RV primers, downstream region was amplified using KT2440_ pyrF _3_FW and KT2440_ pyrF _4_RV primers, kanamycin casette was amplified using FRT-PGK-gb2-neo-FRT_RV and FRT-PGK-gb2-neo-FRT_FW primers. | This study |
| pUC19_SacB_thiI_mut | Suicide mutagenesis vector coding for *thiI* flanking region and a kanamycin casette. Upstream region was amplified using KT2440_thiI_1_FW and KT2440_ thiI _2_RV primers, downstream region was amplified using KT2440_ thiI _3_FW and KT2440_ thiI _4_RV primers, kanamycin casette was amplified using FRT-PGK-gb2-neo-FRT_RV and FRT-PGK-gb2-neo-FRT_FW primers. | This study |
| pUC19_ΔDUF523_seq | Vector used for the sequencing of the *tudS* knockout site surrounding region. Insert was amplified using KT2440_DUF_1_FW and KT2440_DUF_4_RV primers. | This study |
| pUC19_pyrF_seq | Vector used for the sequencing of the *pyrF* knockout site surrounding region. Insert was amplified using KT2440_pyrF_1_FW and KT2440_pyrF_4_RV primers. | This study |
| pUC19_thiI_seq | Vector used for the sequencing of the *thiI* knockout site surrounding region. Insert was amplified using KT2440_thiI_1_FW and KT2440_thiI_4_RV primers. | This study |
| pLATE11-*tudS_A* | Gene was amplified using TudS_A_11_31_FW and TudS_A_11_52_RV primers and cloned using aLICator LIC Cloning and Expression System (Thermo Fisher Scientific, USA) | This study |

**Table S4. Oligonucleotide primers used in this study.**

| Primer name | Sequence 5'->3' | Purpose |
|---|---|---|
| KT2440_pyrF_1_FW | TGGCCTACCGCGAGGCCAATGGGCC | Amplification of 5' flanking region of *pyrF* target gene in *P. putida* KT2440 |
| KT2440_pyrF_2_RV | GGCATCGGTGGTTCGGCACAGGCCCGGCAGGCGGACATGGGCAGGGTCTC | |
| KT2440_pyrF_3_FW | GGGCCTGTGCCGAACCACCGATGCC | Amplification of 3' flanking region of *pyrF* target gene in *P. putida* KT2440 |
| KT2440_pyrF_4_RV | GGCCGCCCTTTAGTGAGGGTTAATTCCTCGATGCCGTGCTGACGCGCATC | |
| KT2440_DUF_1_FW | CGCACTTTCTGCCTGCCGACCTGGT | Amplification of 5' flanking region of *tudS_KT* target gene in *P. putida* KT2440 |
| KT2440_DUF_2_RV | CACCCGCGAAAGGGCCGGCACAGGTTCAATCAGAACGGGTCATTGCCACG | |
| KT2440_DUF_3_FW | ACCTGTGCCGGCCCTTTCGCGGGTG | Amplification of 3' flanking region of *tudS_KT* target gene in *P. putida* KT2440 |
| KT2440_DUF_4_RV | GGCCGCCCTTTAGTGAGGGTTAATTGAGCTGATCGACGAAATCGCCGACC | |
| KT2440_thiI_1_FW | CGCGACGGCATGGTCACGTGGTGCT | Amplification of 5' flanking region of *thiI* target gene in *P. putida* KT2440 |
| KT2440_thiI_2_RV | ACTGCCGCCATACAGCCCCGGCGTCTGGTTAACAGCGCGCGCAGGGCCTG | |
| KT2440_thiI_3_FW | GACGCCGGGGCTGTATGGCGGCAGT | Amplification of 3' flanking region of *thiI* target gene in *P. putida* KT2440 |
| KT2440_thiI_4_RV | GGCCGCCCTTTAGTGAGGGTTAATTCAGCTGTTCGTCGGTGGCACCGAGG | |
| FRT-PGK-gb2-neo-FRT_RV | TAATACGACTCACTATAGGGCTCGA | Amplification of kanamycin resistance cassette from FRT-PGK-gb2-neo-FRT PCR-template (Gene Bridges, Germany) |
| FRT-PGK-gb2-neo-FRT_FW | AATTAACCCTCACTAAAGGGCGGCC | |
| PP_A_11_31_FW | AGAAGGAGATATAACTATGCAGAAAATTCTGGTGAGTCGCTGC | Amplification of *tudS_P* gene for cloning into pLATE11 and pLATE31 vectors |
| PP_A_11_52_RV | GGAGATGGGAAGTCATTAGCCCGCCTCGATTGTTCC | |
| PP_A_31_RV | GTGGTGGTGATGGTGATGGCCGCCCGCCTCGATTGTTCC | |
| TudS_A_11_31_FW | AGAAGGAGATATAACTATGAAGGAAAAAATCATAGTCAGCG | Amplification of *tudS_A* gene for cloning into pLATE11 vector |
| TudS_A_11_52_RV | GGAGATGGGAAGTCATTATCAGGAGCCCGCTGC | |

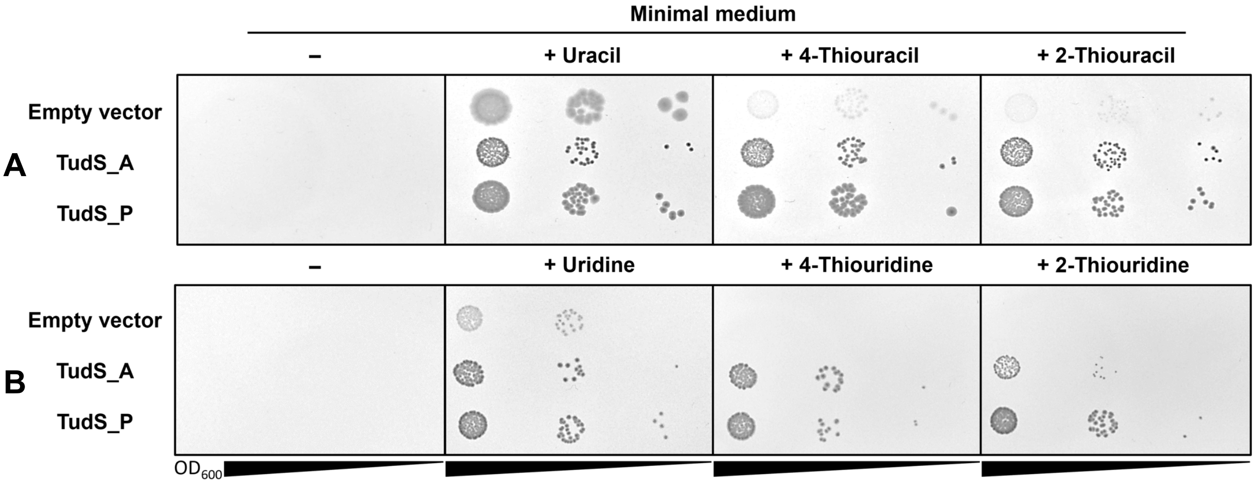


**Figure S1. Growth of the uracil auxotrophic *E. coli* HMS174Δ*pyrF* strain producing TudS_A or TudS_P in the presence of (thio)uracil (A) or (thio)uridine (B) compounds.** The empty pET21b(+) vector served as a negative control. Minimal M9 medium was supplemented as indicated; (thio)uracils and (thio)uridines were added at 20 µg $mL^{-1}$ and 40 µg $mL^{-1}$ respectively.

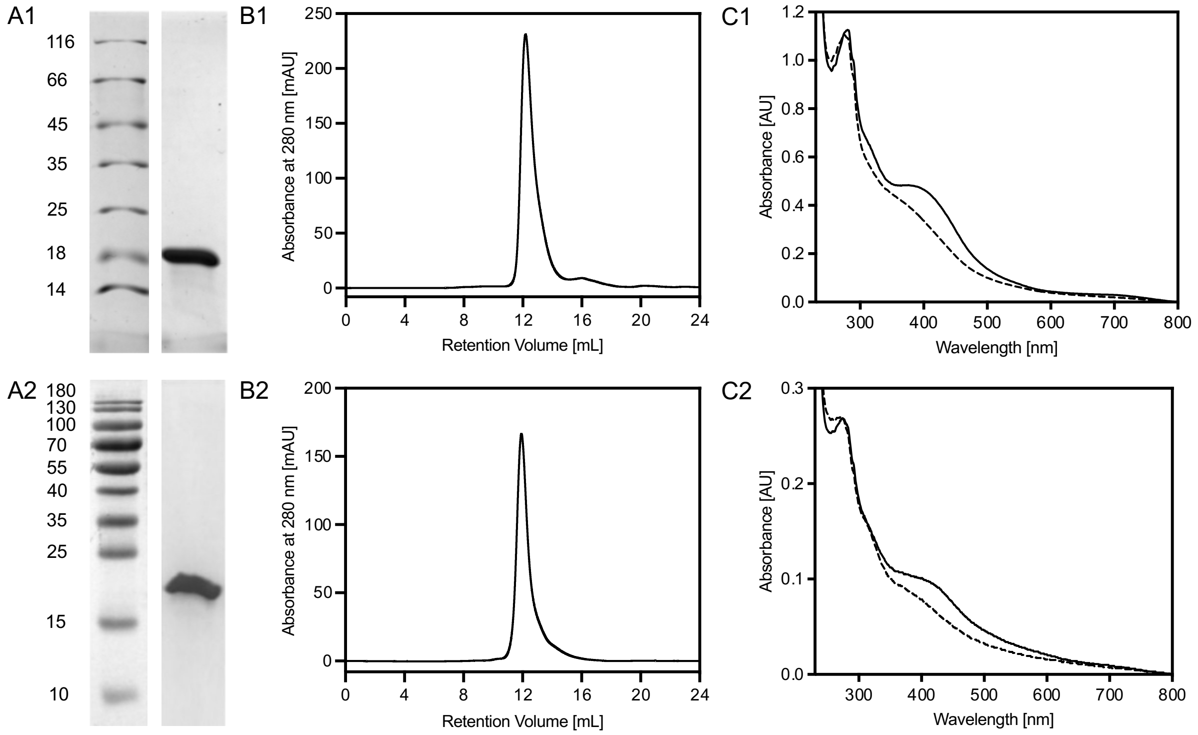


**Figure S2. Analysis of TudS proteins after heterologous production and anaerobic purification. A,** SDS-PAGE analysis of the elution fraction of TudS_A after Ni-NTA chromatography (A1) and the elution fraction of TudS_P after Co-NTA chromatography (~8 µg, respectively; A2). **B,** Analysis of purified TudS_A (B1) and TudS_P (B2) after Superdex 75 10/300 GL gel filtration chromatography. **C,** UV-visible spectra of TudS_A (C1; bold line) and after reduction with dithionite at pH 8 (C1; dotted line). UV-visible spectra of TudS_P (C2; bold line) and after reduction with dithionite at pH 7.3 (C2; dotted line).

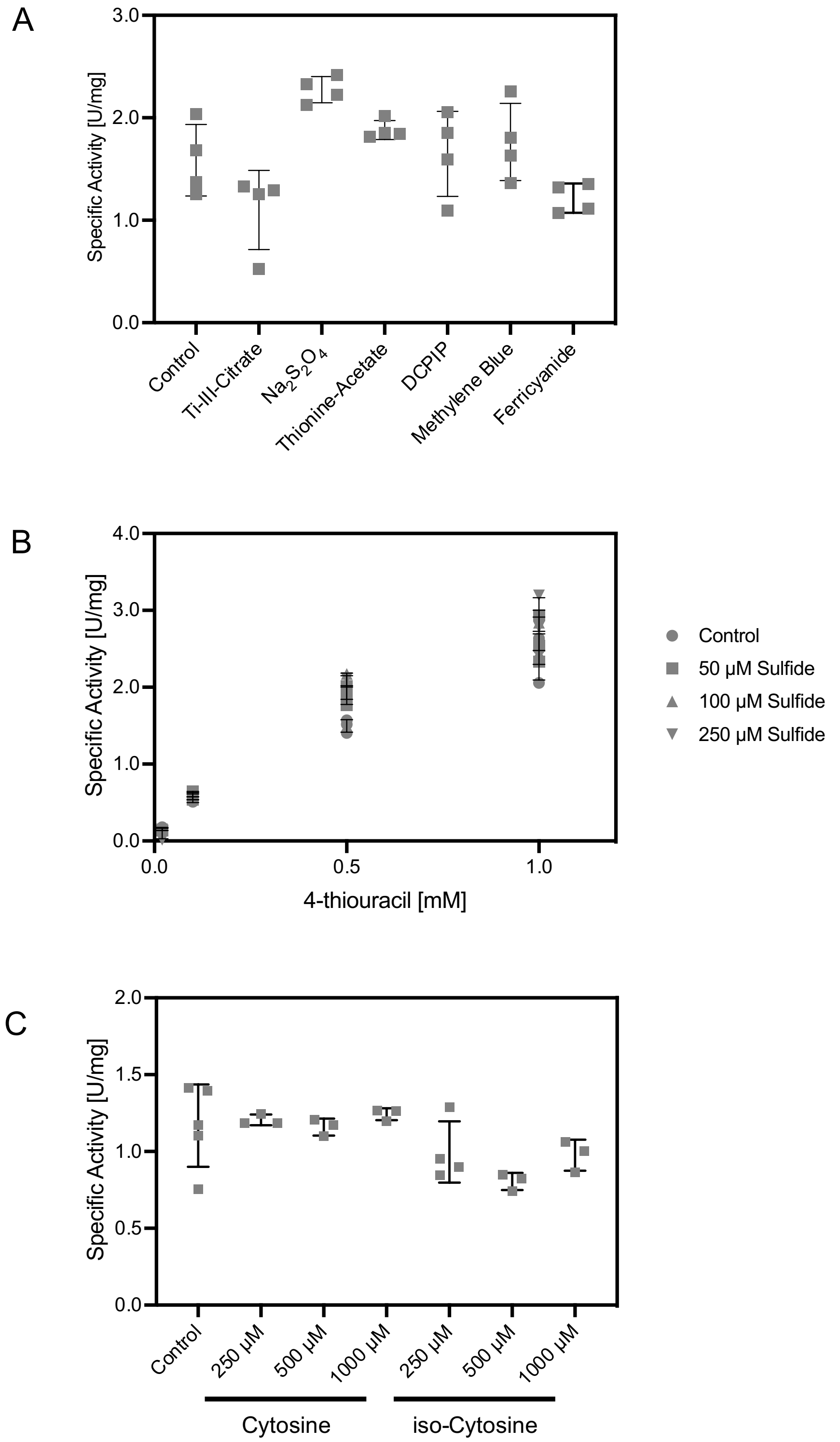


**Fig. S3. Effect of different electron donors or acceptors sulfide, cytosine, and isocytosine on TudS_A activity.** **A,** conversion of 4-thiouracil (0.5 mM) by TudS_A in the presence of indicated redox agents (5 mM, n=4, SD). **B,** conversion of 4-thiouracil (0.5 mM) by TudS_A in the presence of sodium sulfide at concentrations as indicated (n=3, except for 1 missing value for control at 100 µM 4-thiouracil, SD); **C,** conversion of 4-thiouracil (0.5 mM) in the presence of cytosine and isocytosine at the concentrations as indicated (n≥3, SD).

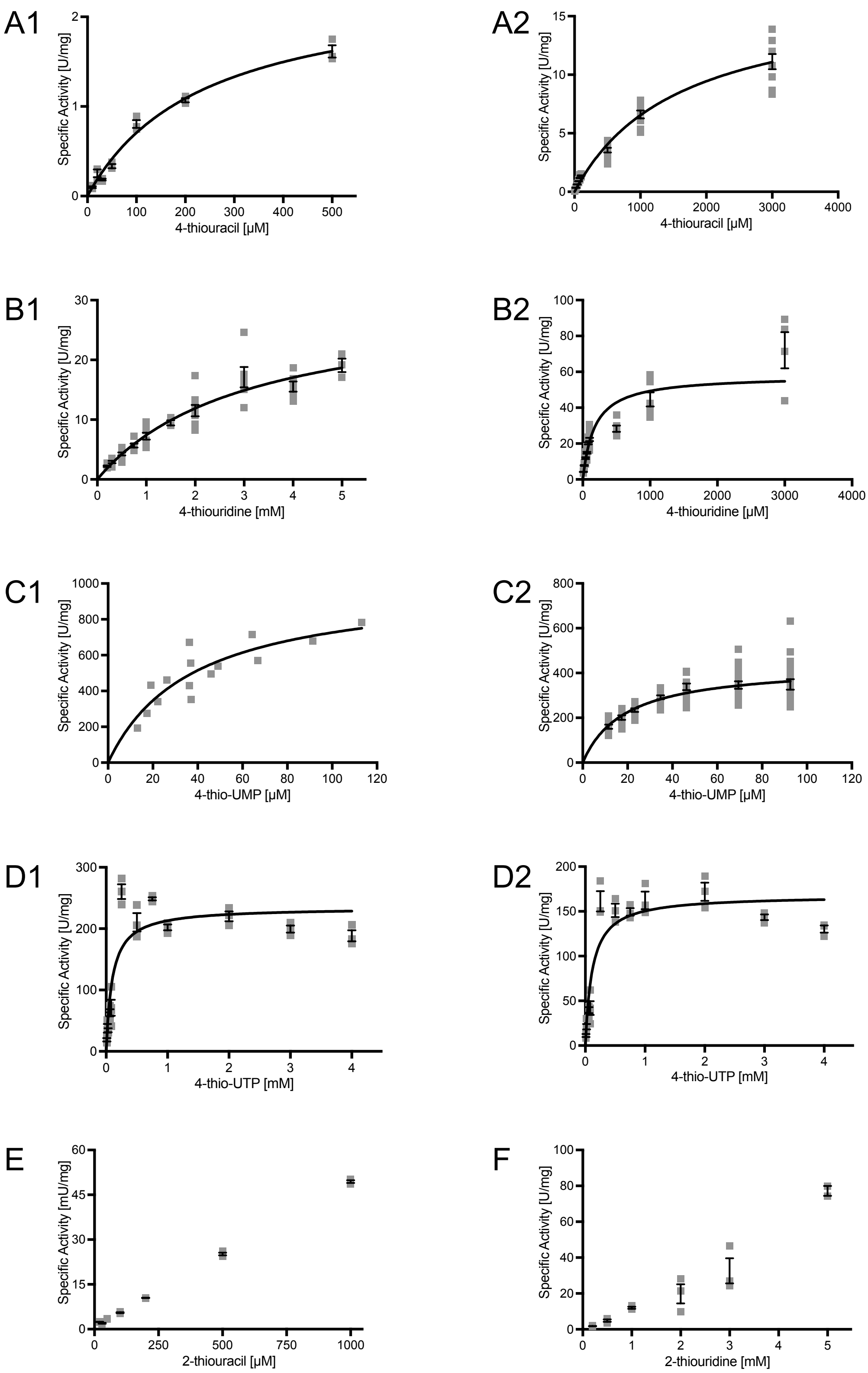


**Figure S4**. **Fit of data obtained for the conversion of 2- and 4-thiouracils, 2- and 4-thiouridine, 4-thiouridine monophosphate and 4-thiouridine triphosphate by TudS_A and/or TudS_P to Michaelis Menten curves.** **A1**, TudS_A + 4-thiouracil (n=3, SE); **A2**, TudS_P + 4-thiouracil (n≥3, SE); **B1**, TudS_A + 4-thiouridine (n≥3, SE); **B2**, TudS_P, 4-thiouridine (n≥3, SE); **C1**, TudS_A + 4-thiouridine monophosphate (4-thio-UMP); **C2**, TudS_P + 4-thio-UMP (n≥3, SE); **D1**, TudS_A + 4-thiouridine triphosphate (4-thio-UTP) (n≥3, SE); **D2**, TudS_P + 4-thio-UTP (n≥3, SE); **E,** TudS_A + 2-thiouracil (n=3, SE); **F**, TudS_A, 2-thiouridine (n=3, SE, 1 outlier removed).

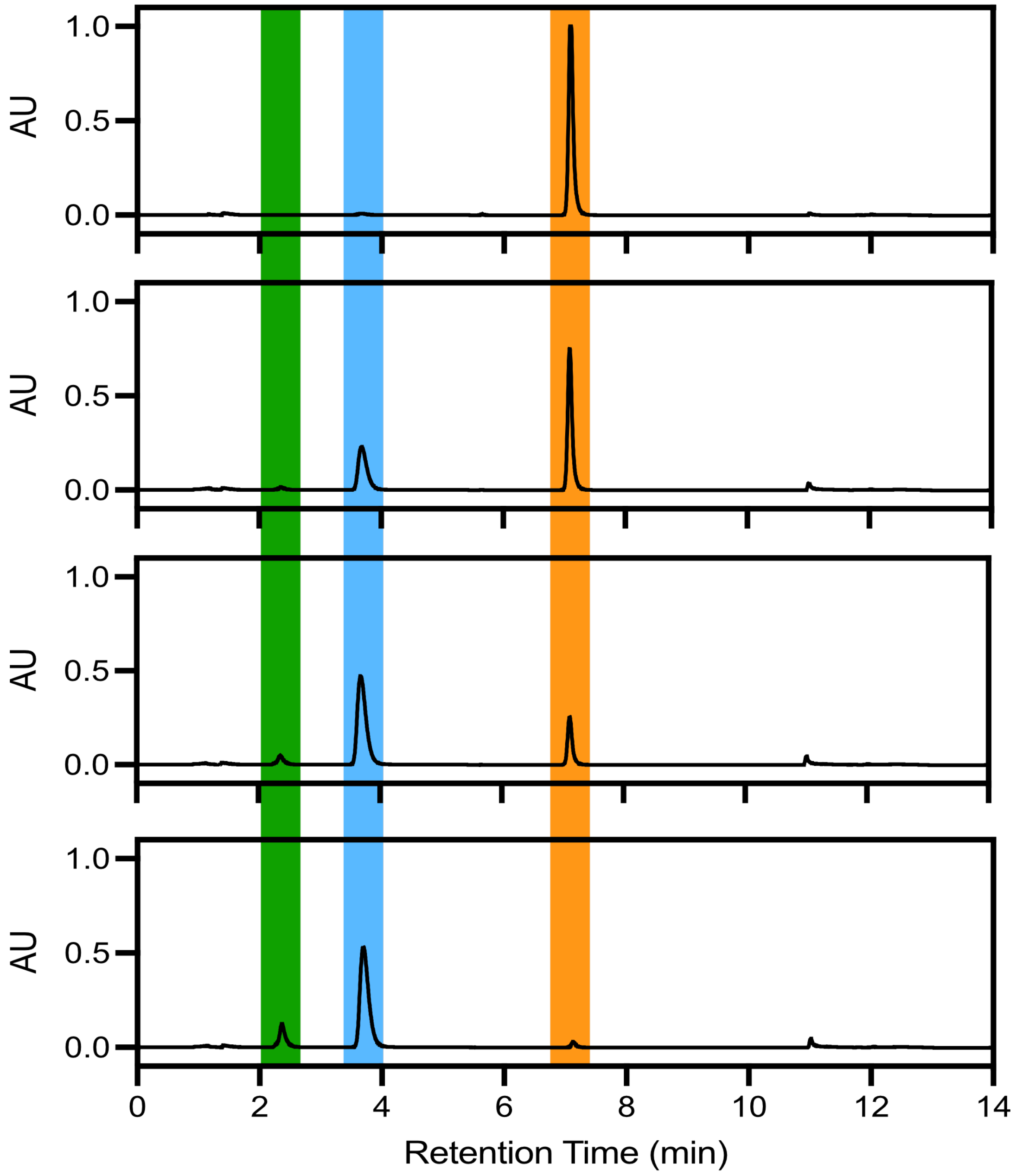


**Fig. S5. UPLC elution diagrams for the conversion of 2,4-dithiouracil by TudS_A.** TudS (50 µM) was incubated with 500 µM 2,4-dithiouracil for 0 min (lane 1), 1 min (lane 2), 5 min (lane 3), and 15 min (lane 4) at 30 °C anaerobically and the substrates/products were analyzed by UPLC. 2,4-thiouracil (orange) is first converted to 2-thiouracil (blue) and then to uracil (green) at a much lower rate. AU = arbitrary units for the diode array detection.

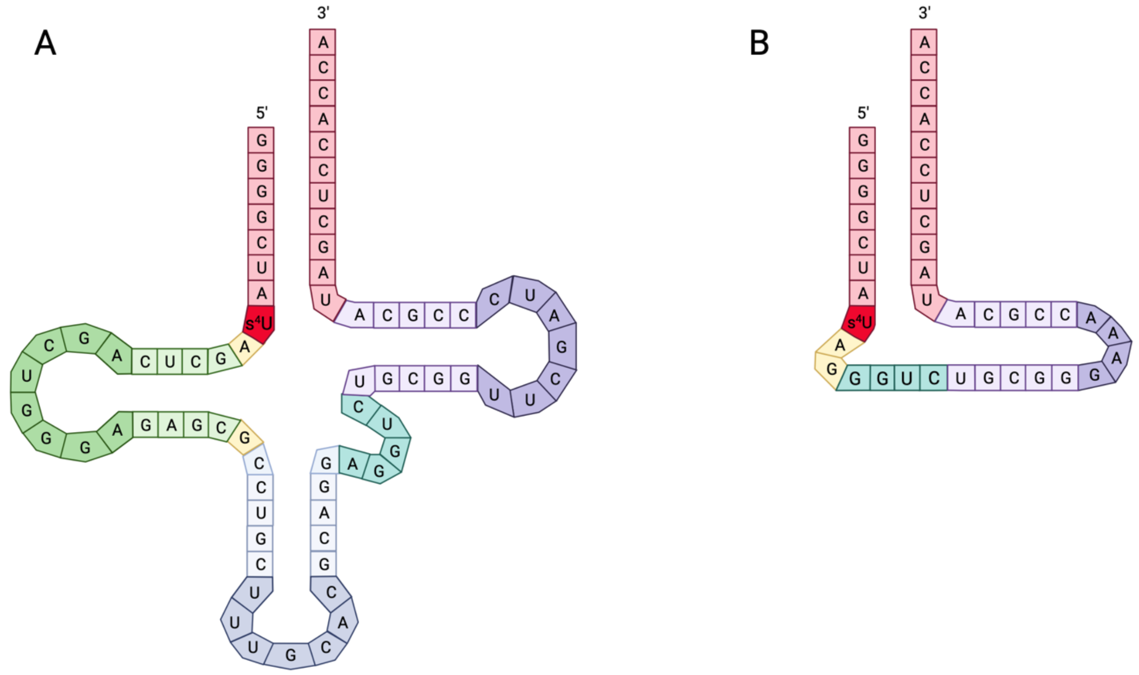


**Fig. S6. Structure of the truncated tRNA tested as possible substrate for TudS enzymes. A**, *Aeromonas hydrophila* tRNA$_{Ala}$ (tRNAdb ID44: tdbD00000071) that was used as a template; **B**, the truncated t-tRNA, consisting of the acceptor stem, the s$^4$U modification at position 8, a shortened T-loop and variable region of the template tRNA.

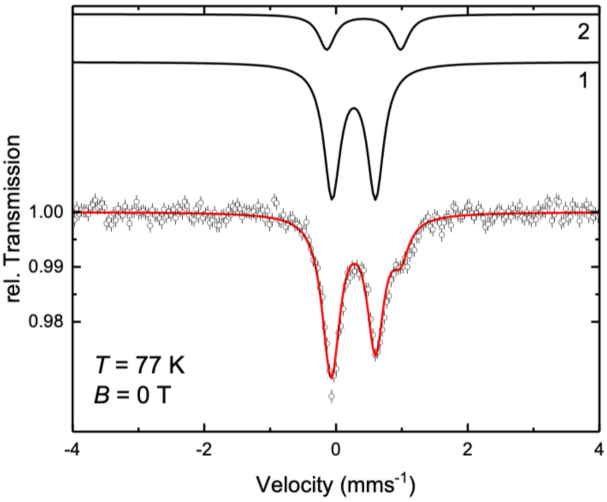


| Components | δ $(\frac{mm}{s})$ | $\Delta E_Q$ $(\frac{mm}{s})$ | Γ $(\frac{mm}{s})$ | Area (%) |
|---|---|---|---|---|
| *1.* $[3Fe4S]^{1+}_{cube}$ | 0.27 (1) | 0.68 (2) | 0.30 (2) | 79 (2) |
| *2.* $[4Fe4S]^{2+}$ | 0.42 (1) | 1.12 (2) | 0.30 (2) | 21 (2) |

**Fig. S7. Mössbauer spectroscopic analysis of TudS_A.** Spectrum of 400 µM TudS_A as isolated. Simulated spectra with the given parameters for a mixture of $[3Fe\text{-}4S]^{+1}$ and $[4Fe\text{-}4S]^{+2}$ were simulated and depicted as red solid lines. Mössbauer conditions: 77K and $B_{ext}$= 0 T.

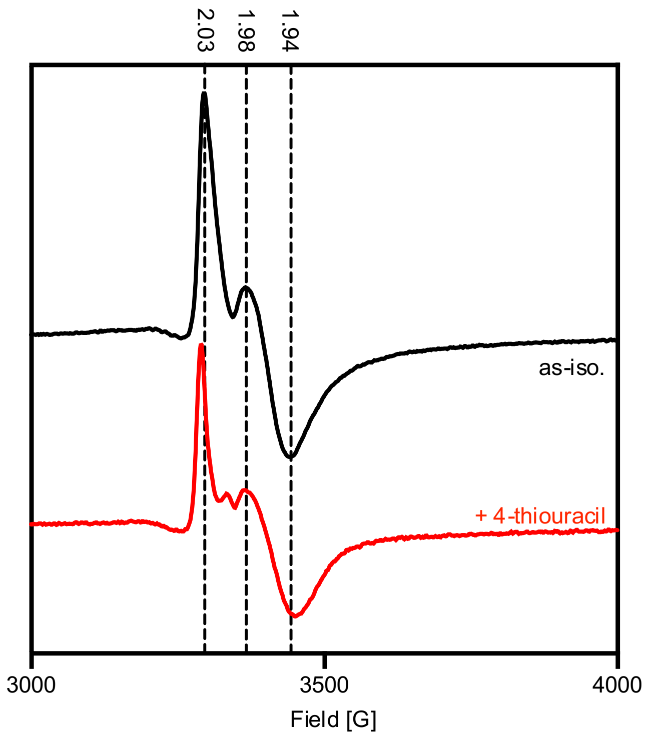


**Figure S8. EPR spectra of as isolated TudS_A at 10 K.** TudS_A without (black) and with 5 mM 4-thiouracil (red). EPR conditions: microwave power, 0.0526 mW; modulation amplitude, 1.5 mT; modulation frequency, 100 kHz.

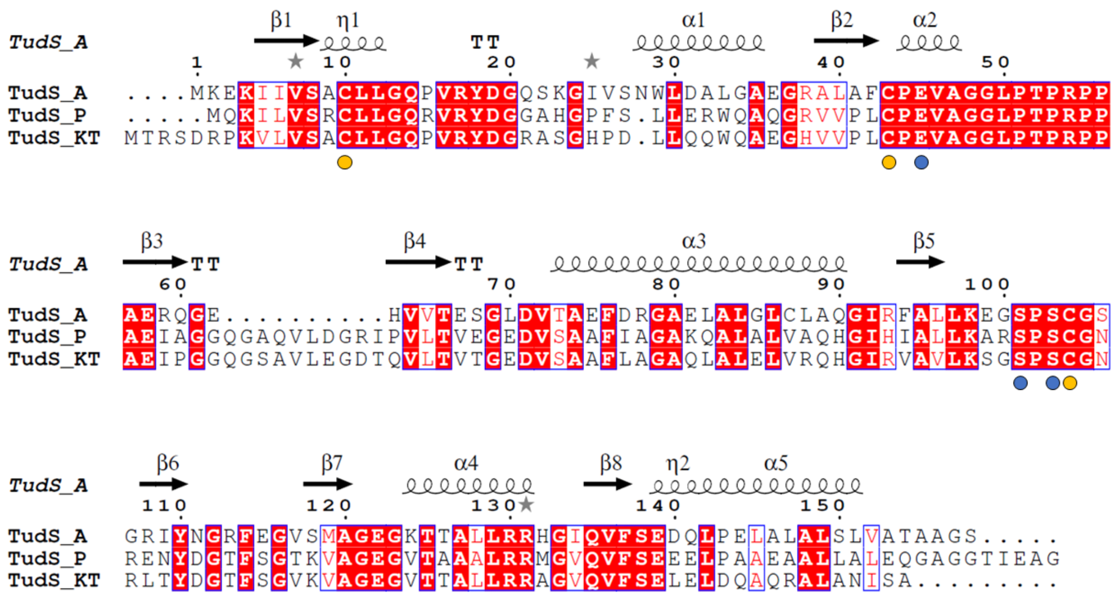

**Figure S9. Sequence alignment of TudS proteins used in this study.** Alignment was performed using Clustal Omega4 and rendered with ESPript[5]. All proteins contain the three conserved cysteines (indicated with yellow dots) that ligate the [4Fe-4S] cluster and the catalytic serine and glutamic acid residues (indicated in blue dots).

**Supplementary References**


1. Akhtar, M. K. & Jones, P. R. Deletion of iscR stimulates recombinant clostridial Fe-Fe hydrogenase activity and H2-accumulation in Escherichia coli BL21(DE3). *Appl Microbiol Biotechnol* **78**, 853–862 (2008).
2. Zhou, J. *et al.* Structural Evidence for a [4Fe-5S] Intermediate in the Non-Redox Desulfuration of Thiouracil. *Angew Chem Int Ed Engl* **60**, 424–431 (2021).
3. Krasauskas, R., Skerniškytė, J., Armalytė, J. & Sužiedėlienė, E. The role of Acinetobacter baumannii response regulator BfmR in pellicle formation and competitiveness via contact-dependent inhibition system. *BMC Microbiol* **19**, 1–12 (2019).
4. Sievers, F. *et al.* Fast, scalable generation of high-quality protein multiple sequence alignments using Clustal Omega. *Mol Syst Biol* **7**, (2011).
5. Gouet, P., Courcelle, E., Stuart, D. I. & Métoz, F. ESPript: analysis of multiple sequence alignments in PostScript. *Bioinformatics* **15**, 305–308 (1999).
