# Supplementary material for: TudS desulfidases recycle 4-thiouridine-5’-monophosphate at a catalytic [4Fe-4S] cluster
Source: Commun Biol. 2023 Oct 27;6:1092. doi: 10.1038/s42003-023-05450-5 (PMC10611767; doi:10.1038/s42003-023-05450-5)
Supplement: Supplementary file 2 — Description of Additional Supplementary Data [file 42003_2023_5450_MOESM2_ESM.docx]

**Description of Additional Supplementary Files**

**File name:** Supplementary Data 1

**Description:** Source data for Figs 2, 4, 5 and Table 1 in the main manuscript; source data for the Figs S2, S4, S5, S7, S8 in Supplemental Material file
